# Two‐Dimensional Boronate Ester Covalent Organic Framework Thin Films with Large Single Crystalline Domains for a Neuromorphic Memory Device

**DOI:** 10.1002/anie.201916595

**Published:** 2020-03-19

**Authors:** SangWook Park, Zhongquan Liao, Bergoi Ibarlucea, Haoyuan Qi, Hung‐Hsuan Lin, Daniel Becker, Jason Melidonie, Tao Zhang, Hafeesudeen Sahabudeen, Larysa Baraban, Chang‐Ki Baek, Zhikun Zheng, Ehrenfried Zschech, Andreas Fery, Thomas Heine, Ute Kaiser, Gianaurelio Cuniberti, Renhao Dong, Xinliang Feng

**Affiliations:** ^1^ Center for Advancing Electronics Dresden (cfaed) & Faculty of Chemistry and Food Chemistry Technische Universität Dresden 01062 Dresden Germany; ^2^ Fraunhofer Institute for Ceramic Technologies and Systems (IKTS) 01109 Dresden Germany; ^3^ Center for Advancing Electronics Dresden (cfaed) & Institute of Materials Science and Max Bergmann Center of Biomaterials Technische Universität Dresden 01069 Dresden Germany; ^4^ Central Facility of Electron Microscopy, Electron Microscopy Group of Materials Science Universität Ulm 89081 Ulm Germany; ^5^ Department of Creative IT Engineering and Future IT Innovation Laboratory Pohang University of Science and Technology (POSTECH) Pohang Korea; ^6^ Key Laboratory for Polymeric Composite and Functional Materials of Ministry of Education Guangdong Engineering Technology Research Center for High-performance Organic and Polymer Photoelectric Functional Films School of Chemistry Sun Yat-Sen University 510275 Guangzhou P. R. China; ^7^ Leibniz-Institut für Polymerforschung Dresden e. V. (IPF) 01069 Dresden Germany; ^8^ Helmholtz-Zentrum Dresden-Rossendorf Institute of Resource Ecology 01328 Dresden Germany

**Keywords:** covalent organic framework, 2D polymer, interfacial synthesis, neuromorphic memory device, single crystal

## Abstract

Despite the recent progress in the synthesis of crystalline boronate ester covalent organic frameworks (BECOFs) in powder and thin‐film through solvothermal method and on‐solid‐surface synthesis, respectively, their applications in electronics, remain less explored due to the challenges in thin‐film processability and device integration associated with the control of film thickness, layer orientation, stability and crystallinity. Moreover, although the crystalline domain sizes of the powder samples can reach micrometer scale (up to ≈1.5 μm), the reported thin‐film samples have so far rather small crystalline domains up to 100 nm. Here we demonstrate a general and efficient synthesis of crystalline two‐dimensional (2D) BECOF films composed of porphyrin macrocycles and phenyl or naphthyl linkers (named as **2D BECOF‐PP** or **2D BECOF‐PN**) by employing a surfactant‐monolayer‐assisted interfacial synthesis (SMAIS) on the water surface. The achieved 2D BECOF‐PP is featured as free‐standing thin film with large single‐crystalline domains up to ≈60 μm^2^ and tunable thickness from 6 to 16 nm. A hybrid memory device composed of 2D BECOF‐PP film on silicon nanowire‐based field‐effect transistor is demonstrated as a bio‐inspired system to mimic neuronal synapses, displaying a learning–erasing–forgetting memory process.

## Graphical abstract

**Mimicking synaptic plasticity**: A free‐standing boronate ester‐linked 2D COF thin film has been achieved with record crystalline domain size as large as ≈60 μm^2^ via an interfacial synthesis method. This film was integrated into an organic thin film/Si nanowire‐based field effect transistor to mimic neuronal synapses capable of learning–erasing–forgetting memory process.

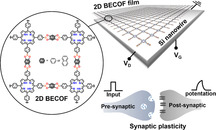

## Introduction

Two‐dimensional boronate ester covalent organic frameworks (2D BECOFs) are known as a class of crystalline, porous polymers with layer‐stacked structures formed by reversible covalent reaction between boronic acid and catechol.^1^ During the last decade, 2D BECOFs have exhibited great potential as active semiconducting layers for (opto‐)electronics,^2^ due to the incorporation of photo‐/electroactive subunits into the backbones, such as pyrene, thiophene, porphyrin and phthalocyanine, that are precisely stacked in a periodic columnar mode. An anisotropic transport was evidenced in the layer‐stacked 2D BECOFs accompanied with an intrinsic electrical conductivity up to ≈10^−7^ S cm^−1^ and a carrier mobility up to ≈1.0 cm^2^ V^−1^ s^−1^ (ac limit by photoconductivity measurements).2b, 2f However, unlike imine‐based 2D conjugated COFs that have already shown promising applications in electronic devices, such as field‐effect transistors^3^ and memristors,^4^ it remains a great challenge to integrate 2D BECOFs into logic and memory devices due to the difficulty in thin‐film processability and device integration associated with the necessity of controlling film thickness, layer orientation, stability and crystallinity.

Currently, a great effort has been dedicated to developing synthetic methodologies toward large‐sized or single‐crystalline COF samples.^5^ Traditionally, solvothermal synthesis of organic crystals is inclined toward to poorly controlled nucleation and aggregation, the resultant COFs are in the form of polycrystalline powders.^6^ Recently, a two‐step approach which separated the nucleation and growth processes was demonstrated, leading to the successful synthesis of single crystalline 2D BECOF powders with domain sizes ranging from 500 nm to 1.5 μm.^7^ On the other hand, bottom‐up on‐solid‐surface synthesis under ultrahigh vacuum condition,^8^ room‐temperature vapor‐assisted conversion^9^ and synthesis on graphene support under solvothermal condition^10^ have been developed for the preparation of various 2D BECOF films from monolayer to micrometer‐thickness. However, these approaches are restricted in terms of small crystalline domains (up to 100 nm) and high defect density, most possibly due to the limited mobility of monomers and random propagation of polymerization. Moreover, the transfer of metal or graphene surface‐binding 2D COF films is a rather complicated issue, which limit the potential device applications.

In this work, we report a novel synthesis of large‐area, crystalline, few‐layer 2D BECOF films composed of porphyrin macrocycles and phenyl or naphthyl linkers (**2D BECOF‐PP** or **2D BECOF‐PN**) utilizing a surfactant‐monolayer‐assisted interfacial synthesis (SMAIS) method.^11^ Anionic surfactant monolayer such as, sodium oleyl sulfate (SOS), was employed on water surface to guide the supramolecular arrangement of C_4_‐symmetric 5,10,15,20‐(tetra‐4‐dihydroxyborylphenyl)porphyrin (**1**) monomers along 2D directions underneath the monolayer. Subsequent polycondensation reaction between monomer **1** and 1,2,4,5‐tetrahydroxybenzene (**2**) or 2,3,6,7‐tetrahydroxynaphthalene (**3**) led to the **2D BECOF‐PP** or **2D BECOF‐PN** thin films. Remarkably, single crystals of **2D BECOF‐PP** with domain size as large as ≈60 μm^2^ could be achieved by this approach, which is much larger than those of thus‐far reported 2D BECOFs (both film and powder samples). The molecular‐level structures are clearly resolved by high‐resolution transmission electron microscopy (HR‐TEM) and selected‐area electron diffraction (SAED) with the support of density functional theory (DFT) calculation. Profiting from the excellent solution processability and mechanical stability of 2D BECOFs from the water surface, for the first time, we integrated the few‐layer **2D BECOF‐PP** film into a silicon nanowire‐based field‐effect transistor (FET), which behaved as a bio‐inspired system to mimic neuronal synapses with a fast response of 20 s for the saturation of the potentiation.

## Results and Discussion

**Figure 1:**
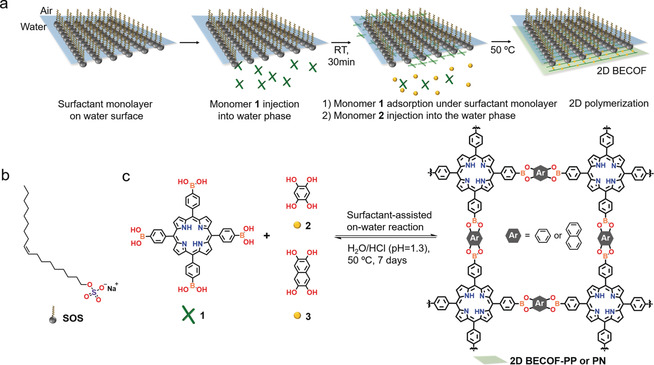
a) Synthesis of boronate ester linked 2D COF films using SMAIS method. b) Chemical structure of the employed anionic surfactant. c) Reaction scheme of 2D BECOFs.

**Figure 2:**
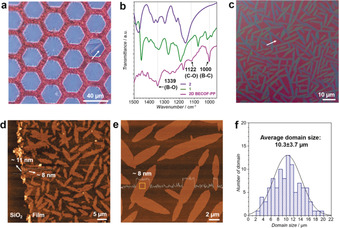
a) Optical microscopy image of **2D BECOF‐PP** film suspended over a copper grid. The white arrow points to a crack in the film. b) FT‐IR spectra of monomers (**1**, **2**) and **2D BECOF‐PP**. c) Polarized microscopy image of **2D BECOF‐PP** film. The white arrow points to amorphous region between domains. d) Atomic force microscopy (AFM) image of film on Si/SiO_2_ substrate. e) Enlarged AFM image of crystalline domains on film. The average roughness on a crystalline domain surface is 0.84 nm (yellow square, 1 μm^2^). f) Single crystal size distribution of **2D BECOF‐PP**.

Figure 1 a illustrates the interfacial synthesis procedure of the targeted 2D BECOFs. Specifically, a chloroform solution of SOS surfactant was dropped onto the water surface and a SOS monolayer was achieved with the RSO_4_^−^ polar head groups facing the water phase after the evaporation of chloroform. Subsequently, 1 mL of acidic aqueous solution comprising monomer **1** (1 μmol) and HCl (1 mmol) was injected into the 40 mL water phase. Due to the electrostatic interactions between protonated porphyrin of monomer **1** 12 and the RSO_4_^−^ head groups of SOS, monomer **1** was readily adsorbed underneath the SOS monolayer (probed by UV/Vis spectroscopy, seen in Figure S1 in the Supporting Information). Next, 1 mL of acidic aqueous solution comprising monomer **2** (4 μmol) and HCl (1 mmol) was added into the water phase and then diffused to the pre‐adsorbed monomer **1**. The mixed solution (pH 1.3) was treated at 50 °C to trigger the polycondensation reaction at the interface. After 7 days, a free‐standing COF film with shiny reflection was observed on the water surface. The resultant **2D BECOF‐PP** film on water was robust enough to be fully transferred onto different substrates for morphological and structural characterizations, such as on Si/SiO_2_ substrate and TEM grid. For instance, the **2D BECOF‐PP** film could suspend over large holes of ≈400 μm^2^ on a TEM grid, which revealed its excellent mechanical stability. (Figure 2 a, Figure S2).

The formation of boronate ester bonds (C_2_O_2_B ring) in the **2D BECOF‐PP** was confirmed by FT‐IR spectroscopy with the appearance of vibrational C−O bonds at 1122 cm^−1^ (Figure 2 b).^13^ Additionally, the strong peak at 1339 cm^−1^ displays the band corresponding to the B−O stretch within the C_2_O_2_B ring. Under the basic condition (such as pH 11.7), we failed to achieve any boronate ester product on the water surface. No C−O bond was detected in the resultant film by FT‐IR (Figure S3). This can be attributed to a rapid oxidation of catechol moieties into quinone in alkaline aqueous solution.^14^ To understand the feasibility of boronate ester formation on water by SAMIS method, we performed the model reaction between monomer **1** and 1,2‐dihydroxybenzene. The reaction was carried out in the acidic aqueous solution (pH 1.3) at 50 °C for 2 days with SOS monolayer. The resultant product on the water surface was analyzed by matrix‐assisted laser desorption/ionization time‐of‐flight mass (MALDI‐TOF MS), which unambiguously confirmed the formation of boronate ester compound with targeted molecular weight (Figure S4). In contrast, the same reaction in aqueous solution (pH 1.3) at 50 °C for 2 days led to negligible conversion to target compound. This result manifested the feasibility of the boronate ester formation on the water surface using SMAIS method.

The morphological features of 2D COF films were studied by polarized microscopy, scanning electron microscopy (SEM) and atomic force microscopy (AFM). The polarized optical image presents ribbon‐like crystal domains with strong bright iridescent colors, revealing long‐range order within these domains, in contrast to the amorphous regions (Figure 2 c). The SEM images also present clear contrast between the crystalline and amorphous regions (Figures S5). The AFM images reveal a thickness of ≈11 nm for the **2D BECOF‐PP** determined by step height from the Si/SiO_2_ substrate (Figure 2 d and Figure S6) while the height profiles present a step height value of the crystalline domains of ≈8 nm from the amorphous area (Figure 2 e and Figure S6). The above morphological observations imply that these individual domains are crystalline and homogeneous. The average size of the resultant crystals was counted as 10.3±3.7 μm (Figure 2 f). Notably, the maximum crystal size even reaches ≈60 μm^2^ (≈18.4 μm × ≈3.3 μm) (Figure S7), which is much larger than those of thus‐far reported 2D BECOFs (up to 1.5 μm).^7^ The areal ratio between the crystalline and amorphous regions is ≈1.12, which suggests that ≈55 % of **2D BECOF‐PP** film is crystalline (Figure S8). It is noted that the average size of single crystals increases upon increasing reaction time (1.0±0.4 μm after 1 day and 4.3±1.1 μm after 4 days, seen in Figure S9). Moreover, the thickness of **2D BECOF‐PP** film could be tuned from 6 to 16 nm (≈8–20 layers) by varying the concentration of monomer **1** from 0.5 μmol to 2.0 μmol and monomer **2** from 2.0 μmol to 8.0 μmol (Figure S10).

**Figure 3:**
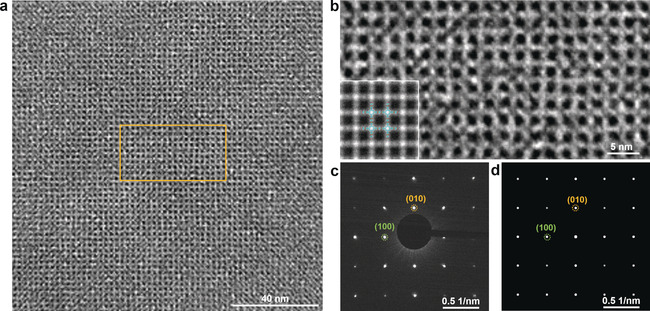
a) HR‐TEM image of **2D BECOF‐PP** film that shows a long‐range ordered 2D networks of square‐patterned pores. b) Enlarged HR‐TEM image from the region in image a (yellow square). Inset is lattice‐averaged image calculated from the DFT. c) Experimental and d) simulated electron‐diffraction patterns, the green and yellow circles indicate the (100) and (010) planes, respectively.

Next, we visualized the molecular structure of **2D BECOF‐PP** single crystalline domains by HR‐TEM (Figure 3 a), which shows long‐range ordered square lattices (Figure 3 a,b). Based on HR‐TEM image simulation (inset of Figure 3 b), the darkest part corresponds to the pores between the porphyrin units (Figure S11) while the bright region shows the square arrangement of the 2D COF backbones. The selected‐area electron diffraction (SAED) pattern (Figure 3 c) displays a square diffraction pattern with nearest reflections corresponding to 0.406 nm^−1^ (i.e., *a*=*b*=2.46 nm, *γ*=90°), which is in line with the AA‐eclipsed stacked atomic model of **2D BECOF‐PP** derived by density functional theory (DFT) calculation (calculated reflection at (100) and (010): 0.402 nm^−1^; Figure 3 d, Figure S12 and Table S1; simulation details seen in SI). In contrast, for AB‐stacking mode (Figure S12 b,d), due to the formation of a body‐centered tetragonal lattice, systematic extinction of (h k 0) reflections (where h + k=odd integers) appears, which can be ruled out from the experimental SAED results (Table S2).

In order to define the crystallinity of **2D BECOF‐PP**, SAED pattern was collected by shifting the condenser aperture (with a diameter of 700 nm) every 2 μm apart along the ribbon‐like crystalline domain (Figure S13). All the diffraction patterns within the rectangle space of 12×4 μm are identical, demonstrating a single‐crystalline structure. We also performed the SAED at various spots of crystals and found that the diffraction patterns were identical, further confirming the single crystal nature for each grain. This result is consistent with the morphological study using polarized microscope and SEM.

To gain insights into the role of surfactants on the interfacial polymerization, we performed the identical reaction protocol without employing surfactant monolayer, which resulted in only amorphous films (Figure S14). We further investigated various surfactant monolayers. Among them, anionic surfactant like sodium dodecylbenzenesulfonate (SDBS) functioned similar to SOS. Cationic, zwitterionic and nonionic surfactants only afforded amorphous films, which can be attributed to the low ordering of monomers under these surfactant monolayers (Figure S15). In order to identify the unique role of the anionic surfactant monolayer, we also investigated the supramolecular structure of porphyrin monomer **1** under the SOS monolayer. The interfacial assembly was carried out under the identical conditions without the addition of monomer **2**. Notably, a free‐standing supramolecular film based on monomer **1** could be obtained. After transferring the supramolecular film onto TEM grids, the SAED patterns manifested a square diffraction pattern with nearest reflections corresponding to 0.454 nm^−1^ (i.e., *a*=*b*=2.20 nm, *γ*=90°), testifying an ordered 2D network with square lattice in supramolecular assembled film of **1**. By contrast, without using surfactant monolayer, it was not possible to obtain such free‐standing film of monomer **1** at the air–water interface (Figure S16).

**Figure 4:**
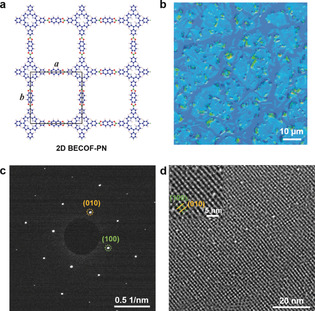
a) Chemical structure of **2D BECOF‐PN** b) Optical microscopy image of **2D BECOF‐PN** film deposited over a Si/SiO_2_ substrate. c) Electron‐diffraction pattern of **2D BECOF‐PN**, the green and yellow circles indicate the (100) and (010) planes, respectively. d) HR‐TEM image of **2D BECOF‐PN**. Inset is the enlarged HR‐TEM image.

Based on the comprehensive understanding of the SMAIS of **2D BECOF‐PP** on the water surface, we extended the synthesis method towards another highly crystalline 2D BECOF composed of porphyrin macrocycles and naphthyl linkers (**2D BECOF‐PN**) and thus proved the generality of the chemistry. The **2D BECOF‐PN** film was synthesized by condensation reaction between monomer **1** and 2,3,6,7‐tetrahydroxynaphthalene (**3**), as shown in Figure 1 c and Figure 4 a. The optical image displays a number of aggregated ribbon‐like crystals with the size ranging from ≈3 to ≈10 μm in the resultant film (Figure 4 b). The areal ratio between the crystalline and amorphous regions is ≈1.56 (Figure S17a). FT‐IR spectrum shows the characteristic bands of C_2_O_2_B ring (Figure S17 b), The SAED of **2D BECOF‐PN** in Figure 4 c reveals a square diffraction pat‐tern with nearest reflections corresponding to 0.384 nm^−1^, (i.e., *a*=*b*=2.60 nm, *γ*=90°), which is well supported by the AA‐eclipsed atomic model obtained from DFT calculation (calculated reflection at (100) and (010): 0.366 nm^−1^, Figure S18). HR‐TEM image further visualizes the molecular structure of **2D BECOF‐PN** crystals and confirms the 2D extended square lattices with the size of 2.60 nm (Figure 4 d).

**Figure 5:**
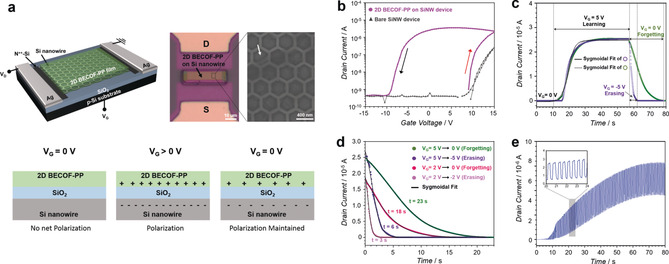
a) Schematics of the **2D BECOF‐PP**/SiNW hybrid device. The white arrow points to a crack in the film on SiNW. b) Transfer characteristics of **2D BECOF‐PP**/SiNW hybrid device. c) Learn–erase/learn–forget cycles. d) History of the input signal in relaxation process. e) Short‐term potentiation with learning pulse.

Benefited from the excellent solution processability by interfacial transfer and the high crystallinity, the orderly‐stacked porphyrin macrocycles in **2D BECOF‐PP** film can generate transverse field with positive charge which is counter‐balanced by the electrons accumulation.^15^ Therefore, we envisage that **2D BECOF‐PP** will provide a pseudo‐gate to emulate the synaptic plasticity with silicon nanowire (SiNW) FET.^16^ To this end, synaptic behavior of **2D BECOF‐PP**/SiNW hybrid device was explored for the first time, with synaptic dynamics at a hardware level compatible with silicon semiconductor processes. The n‐type honeycomb silicon nanowire FET was prepared by electron‐beam lithography^17^ for hybrid device. A resultant **2D BECOF‐PP** film with thickness of 11 nm was transferred via vertical deposition method onto SiNW device to accumulate the surface charge at the **2D BECOF‐PP** film/SiO_2_ interface under the positive input gate voltage (*V*_G_). The surface charge was maintained by the accumulated charges (residual polarization) when the bias returned to 0 V, thus causing the memory effect (Figure 5 a). Substantial hysteresis, manifesting about the charge trapping and storage capability of the device, was observed for hybrid device. The threshold voltage was higher in the up‐sweep (red arrow in Figure 5 b) stage than in the down‐sweep (black arrow in Figure 5 b) stage, suggesting the positive polarity of the trapped charges in the device, while the threshold voltage of bare device was constant. This hysteresis behavior is compulsory for the memory effects, including potentiation (learning), depression (erasing) and relaxation process (forgetting). The **2D BECOF‐PP**/SiNW hybrid device presents a fast response of ≈20 s for the saturation of the potentiation (Figure 5 c). Figure 5 d shows that depression time constant in the forgetting period (23 s, *V*_G_=5 V→0 V) extended more than triple the time constant in the erasing period (6 s, *V*_G_=5 V→−5 V). In contrast, **2D BECOF‐PP** film alone as well as bare SiNW device could not afford hysteresis loop and potentiation (Figure S19). Based on the understanding of memory effect in **2DBECOF‐PP**/SiNW hybrid device, we next applied synaptic dynamics by modulating 5 V pulses with the same period (500 ms) and duty cycle (500 ms) for neuroinspired behavior (Figure S20). Resultant clear short‐term potentiation presents a history‐dependent memory, as a key intrinsic feature of neuron, which is stored as ionic state in the **2D BECOF‐PP** film (Figure 5 e). Thereby, our approach using 2D BECOF thin film/SiNW hybrid device allowed an emulating the neuronal membrane intrinsic plasticity.

## Conclusion

In summary, we demonstrated an efficient synthesis of free‐standing, crystalline boronate ester 2D COF thin films with tunable thickness from 6 to16 nm via the SMAIS method. The single‐crystalline domain size in **2D BECOF‐PP** thin film reached as large as ≈60 μm^2^, which is superior to those of reported 2D BECOFs. Due to the high crystallinity, facile thin‐film processability, high mechanical stability as well as the incorporation of electroactive porphyrin monomers, the developed few‐layer **2D BECOF‐PP** film was for the first time integrated into an organic thin film/SiNW‐based FET to mimic neuronal synapses. Such artificial synaptic transistor displayed a learning–erasing–forgetting memory process with a fast response of ≈20 s for the saturation of the potentiation. Our work broadens the interfacial synthesis of highly crystalline, few‐layer 2D COF or 2D polymer thin films and opens up a new area for developing such emergent materials as active components in memory devices for future neuromorphic computing, which also provides possibilities for the future development of COF‐based flexible and wearable logic and memory electronics.

## Conflict of interest

The authors declare no conflict of interest.

## Supporting information

As a service to our authors and readers, this journal provides supporting information supplied by the authors. Such materials are peer reviewed and may be re‐organized for online delivery, but are not copy‐edited or typeset. Technical support issues arising from supporting information (other than missing files) should be addressed to the authors.

Supplementary
