# Supplementary material for: Two‐Dimensional Boronate Ester Covalent Organic Framework Thin Films with Large Single Crystalline Domains for a Neuromorphic Memory Device
Source: Angew Chem Int Ed Engl. 2020 Mar 19;59(21):8218–24. doi: 10.1002/anie.201916595 (PMC7317805; doi:10.1002/anie.201916595)
Supplement: Supplementary file 1 — Supplementary [file ANIE-59-8218-s001.pdf]

Angewandte Chemie
Eine Zeitschrift der Gesellschaft Deutscher Chemiker

Supporting Information

Two-Dimensional Boronate Ester Covalent Organic Framework Thin Films with Large Single Crystalline Domains for a Neuromorphic Memory Device

SangWook Park, Zhongquan Liao, Bergoi Ibarlucea, Haoyuan Qi, Hung-Hsuan Lin, Daniel Becker, Jason Melidonie, Tao Zhang, Hafeesudeen Sahabudeen, Larysa Baraban, Chang-Ki Baek, Zhikun Zheng, Ehrenfried Zschech, Andreas Fery, Thomas Heine, Ute Kaiser, Gianaurelio Cuniberti, Renhao Dong,* and Xinliang Feng**

anie_201916595_sm_miscellaneous_information.pdf

Table of Contents

Materials and Methods……3
- Materials……3
- Methods……3
- Domain size of 2D BECOF-PP……3
- Single crystalline domain size of 2D BECOF-PP determined by SAED……3
- High magnification TEM images……3
- TEM image simulation and SAED simulation……3
- Structural modelling……3
- Neuromorphic memory device fabrication……4
- Neuromorphic memory device measurement……4

Synthetic Procedures……5
- Synthesis of 5,10,15,20-(Tetra-4-dihydroxyborylphenyl)porphyrin……5
- Synthesis of 1,2,4,5-tetrahydroxybenzene……5
- Synthesis of 2,3,6,7-tetrahydroxynaphthalene……5
- Synthesis of 2D BECOF-PP film……5
- Synthesis of 2D BECOF-PN film……5
- Synthesis of self-assembled film of monomer 1……6
- Model reaction……6

Figures and Tables……7
- Figure S1……7
- Figure S2……8
- Figure S3……9
- Figure S4……10
- Figure S5……11
- Figure S6……12
- Figure S7……13
- Figure S8……14
- Figure S9……15
- Figure S10……16
- Figure S11……17
- Figure S12……18
- Figure S13……20
- Figure S14……21
- Figure S15……22
- Figure S16……23
- Figure S17……24
- Figure S18……25
- Figure S19……27
- Figure S20……27
- Table S1……19
- Table S2……19
- Table S3……26
- Table S4……26
- Table S5……26

Reference……28

Materials and Methods

Materials

Sodium oleyl sulfate (SOS), sodium dodecylbenzenesulfonate (SDBS), 3-(Decyldimethylammonio)propanesulfonate (DAPS), cetrimoniumbromide (CTAB), lignoceryl alcohol (LA) and 1,2-dihydroxybenzene were purchased from Sigma-Aldrich (Germany). 2,3,6,7-Tetramethoxynaphthalene was purchased from Matrix Scientific (USA). 4-(1,3,2-Dioxaborinan-2-yl)benzaldehyde was purchased from Fisher Scientific (Germany). All compounds were used as received.

Methods

Optical images were acquired in reflected-light mode or reflected-light mode under polarizer with AxioScope A1 (Carl Zeiss) The scanning electron microscopy (SEM) images were recorded on a field emission scanning electron microscope (FESEM, Carl Zeiss Gemini) operating at an accelerating voltage of 3 kV. High magnification transmission electron microscopy (TEM) was performed using a LIBRA 200 MC Cs STEM (Carl Zeiss) operating at an accelerating voltage of 200 kV. Atomic force microscopy (AFM) was performed in air on a customized Ntegra Aura/Spectra from NT-MDT (Moscow, Russia) with a SMENA head operated in contact mode. Fourier-transform infrared spectroscopy (FTIR) was performed on Tensor II (Bruker) with an attenuated total reflection (ATR) unit. UV-Vis absorption spectra were obtained on an UV-Vis-NIR Spectrophotometer Cary 5000 at room temperature. Mass spectrometry analysis was performed on a Bruker Autoflex Speed MALDI TOF MS (Bruker Daltonics, Bremen, Germany) using dithranol as matrix. NMR data were recorded on a Bruker AV-II 300 spectrometer operating at 300 MHz for ^{1}H and 75 MHz for ^{13}C with standard Bruker pulse programs at room temperature (296 K). Chemical shifts were referenced to δ_{TMS} = 0.00 ppm (^{1}H, ^{13}C). Chemical shifts (δ) are reported in ppm. Dimethylsulfoxide-d$_6$ (δ(^{1}H) = 2.50 ppm, δ(^{13}C) = 39.52 ppm) was used as solvent. The following abbreviations are used to describe peak patterns as appropriate: s = singlet, d = doublet, t = triplet, q = quartet, and m = multiplet. Dimethylsulfoxid-d$_6$ (99.96 atom% D) was purchased from Deutero GmbH.

Domain size of 2D BECOF-PP

The size distribution of **2D BECOF-PP** domains were characterized using optical microscope (OM) images. The sizes of isolated 100 domains from multiple OM image for each reaction time were measured manually using ImageJ, always in the longer horizontal direction through the center of the domain in order that shape anisotropies do not skew the distribution results. In case of 7days reaction of **2D BECOF-PP,** longer horizontal direction (x axis) as well as shorter vertical direction (y axis) were measured, accordingly area of domain was calculated as cross product of both values. Due to the fact that, it is assumed the radial or cross shaped grain might introduce any orientation anisotropy, only isolated rice shaped domains were counted.

Single crystalline domain size of 2D BECOF-PP determined by SAED

The SAED of the synthesized **2D BECOF-PP** was acquired in the TEM using a condenser aperture with a diameter of about 700 nm. The illumination step was set to the lowest step 1, in order to minimize the beam damage on the sample. The exposure time for the each SAED was 1s. SAEDs were sequentially recorded along one certain axis only using goniometer to shift the sample every 2 µm apart. SAEDs from two vertical axes were acquired to estimate the dimension of a single crystalline **2D BECOF-PP** domain.

High resolution TEM images

All high magnification TEM images were recorded using a quasi-blind method since the film is too sensitive to the electron beam in the TEM. Very short exposure (less than 1s) under the electron beam above illumination step 10 was able to destroy/modify the crystalline structure of the sample completely. All parameters (illumination step, focus, astigmatism correction etc.) were optimized first, then electron beam was shut down. The sample was shifted by goniometer blindly, and was settled down for about 2 min. The TEM image was recorded simultaneously while opening the electron beam.

TEM image simulation and SAED simulation

HRTEM image simulation was carried out using multi-slice algorithm in QSTEM software. SAED pattern simulation was conducted using Bloch wave algorithm in JEMS software.

Structural modelling

Density functional theory (DFT) calculations were done using CP2K package.[1] Exchange-correlation functional was treated with a revised Perdew-Burke-Ernzerhoff functional for solids and surfaces (PBEsol)[2] and dispersion was corrected by the DFT-D3 method.[3] All electron calculation with 6-31G* basis sets were used for the monolayer and the multilayer. To model the monolayer of 2D polymer

we added a vacuum space of 20 Å in the z-direction to avoid spurious interactions between layers. Monkhorst-Pack **k**-points mesh for the Brillouin zone was employed with $4 \times 4 \times 1$ and $4 \times 4 \times 8$ for the monolayer and multilayer respectively. During the structural relaxation the convergence thresholds for the maximum force component and the root mean square force were 10^{-5} hartree/bohr and 3×10^{-4} hartree/bohr respectively.

Neuromorphic memory device fabrication

Silicon nanowire-based field-effect transistors (FETs) were fabricated, as reported in previous publications[4,5] , on 200 nm-thick oxide and 40 nm-thick top silicon wafers. The electrode area was doped by a heavy implantation of arsenic and rapid thermal annealing at 1000 °C, followed by inductively coupled plasma reactive ion etching in order to isolate the active region including electrode and channel area. Electron beam lithography process was used to define the honeycomb structure of the channel area, formed by interconnected 50 nm-wide wires for better mechanical stability. Stacked layers of titanium (50 nm) and silver (200 nm) were evaporated to form the electrodes and contact pads. 4 nm thermal oxide was grown in a thermal furnace at 850 °C and finally the device was isolated by a spin-coated 2 µm-thick SU-8 negative tone resist, with the exception of the nanowires which were left exposed for interaction with the **2D BECOF-PP** film. The **2D BECOF-PP** film was deposited on the honeycomb area by vertical transfer from water surface, then dried 6 hours under vacuum.

Neuromorphic memory device measurement

The transfer characteristics of the device and the current vs. time measurements at constant DC voltage were measured using a 2604B Keithley source measure unit (Tektronix GmbH, Germany). The pulsed measurements were carried out by using an AFG 1062 function generator (Tektronix GmbH, Germany) as gate voltage supply. The complete transfer characteristic of the transistor was measured with the applied gate sweeping from -15 V to15 V at the drain voltage V_D = 0.1 V.

Synthetic Procedures

Synthesis of 5,10,15,20-(Tetra-4-dihydroxyborylphenyl)porphyrin

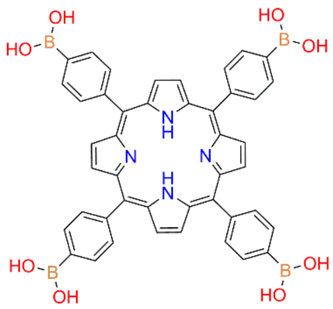

1

The synthesis was adopted reported literature procedures.[6,7] 5,10,15,20-tetrakis(4-(1,3,2-dioxaborinan-2-yl)phenyl)porphyrin (13 mg, 0.013 mmol, 1 eq) was added to a mixture of THF (4 mL), H_2O (1 mL) and conc. HCl (0.25 mL) in a 25 mL Schlenk flask. The reaction mixture was stirred at room temperature. After 20 hours, 10 mL of water were added to the reaction and the precipitate was filtered off and washed carefully with water (50 mL) and chloroform (25 mL). The product was dried under reduced pressure to give 5,10,15,20-(Tetra-4-dihydroxyborylphenyl)porphyrin (8 mg, 0.010 mmol) as green solid in 74% yield.
^{1}H NMR (300 MHz, dimethylsulfoxide-d$_6$, δ): 8.83 (s, 8 H, -CH), 8.22 (q, 16 H, -CH), -2.88 (s, 2 H, -NH) ppm. The peak for -B(OH)$_2$ was not observed due to the broadening of the signal.
^{13}C NMR (75 MHz, dimethylsulfoxide-d$_6$, δ): 142.8, 133.5, 132.6, 120.2 ppm.
MS (MALDI-TOF): m/z = 789.9735, calculated for $C_{44}H_{34}B_4N_4O_8$: m/z = 790.2749.

Synthesis of 1,2,4,5-tetrahydroxybenzene

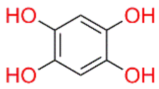

2

The synthesis was adapted from the literature procedure.[8] After filtration and washing with THF the crude solid was recrystallized two times from THF to give the target compound as off-white solid in 59.2% yield.
^{1}H NMR (300 MHz, dimethylsulfoxide-d$_6$, δ): 7.97 (s, 4 H, -OH), 6.20 (s, 2 H, -CH) ppm.

Synthesis of 2,3,6,7-tetrahydroxynaphthalene

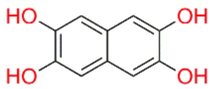

3

The synthesis was adapted from the literature procedure.[9] The crude compound was dissolved in acetonitrile and was purified additionally by column chromatography with pure acetonitrile as solvent to give the target compound as purple solid in 25.8% yield.
^{1}H NMR (300 MHz, dimethylsulfoxide-d$_6$, δ): 8.89 (s, 4 H, -OH), 6.79 (s, 4 H, -CH) ppm.
^{13}C NMR (75 MHz, dimethylsulfoxide-d$_6$, δ): 144.35, 123.76, 108.38 ppm.

Synthesis of 2D BECOF-PP film

Solution preparation: 1 mg of sodium oleyl sulfate (SOS) was dissolved in 1 mL chloroform. Monomer **1** (0.790 mg, 1 μmol) was dissolved in 1 mL of HCl (1 M). Monomer **2** (0.567 mg, 4 μmol) was dissolved in 1 mL of HCl (1 M) and sonicated for 5 min.
Polymerization reaction: 20 μL of SOS/chloroform solution was spread onto the surface of 40 mL Milli-Q water in a 60 mL beaker. The chloroform solvent was allowed to evaporate from water surface for 10 min, then 1 mL of monomers **1** solution was injected into the water phase. After 30 min, 1 mL of monomers **2** solution was injected into the water phase. The beaker was left into an oven at 50 °C for 7 days.

Synthesis of 2D BECOF-PN film

Solution preparation: 1 mg of sodium oleyl sulfate (SOS) was dissolved in 1 mL chloroform. Monomer **1** (0.790 mg, 1 μmol) was dissolved in 1 mL of HCl (1 M). Monomer **3** (0.767 mg, 4 μmol) was dissolved in 1 mL of HCl (1 M) and sonicated for 5 min.
Polymerization reaction: 20 µL of SOS/chloroform solution was spread onto the surface of 40 mL Milli-Q water in a 60 mL beaker. The chloroform solvent was allowed to evaporate from water surface for 10 min, then 1 mL of monomers **1** solution was injected into the water phase. After 30 min, 1 mL of monomers **3** solution was injected into the water phase. The beaker was left into an oven at 50 °C for 7 days.

Synthesis of self-assembled film of monomer 1

Solution preparation: 1 mg of sodium oleyl sulfate (SOS) was dissolved in 1 mL chloroform. Monomer **1** (0.790 mg, 1 μmol) was dissolved in 2 mL of HCl (1 M) and sonicated for 1 hour.
Polymerization reaction: 20 µL of SOS/chloroform solution was spread onto the surface of 40 mL Milli-Q water in a 60 mL beaker. The chloroform solvent was allowed to evaporate from water surface for 10 min, then 2 mL of monomer **1** solution was injected into the water phase. After 30 min, the beaker was left into an oven at 50 °C for 7 days.

Model reaction

On water surface: 3 µL of SOS/chloroform solution was spread onto the surface of 10 mL Milli-Q water in a 20 mL scintillation vial. Monomer **1** (5 μmol) in 1M HCl aqueous solution was injected into the water phase. After 30min, 1,2-dihydroxybenzene in 1M HCl aqueous solution was injected into the water phase. This reaction vial was heated without stirring at 50 °C for 2 days. The products on water surface were fished out, and then immediately measured by MALDI TOF MS and FT-IR
In aqueous solution: Monomer **1** (5 μmol) and 1,2-dihydroxybenzene (20 μmol) in a mixture of Milli-Q water: 1M HCl (20:1 by volume; 10 mL) were sonicated for 1 hour. This solution was put into a 20 mL scintillation vial and sealed. This reaction mixture was heated without stirring at room temperature or 50 °C for 2 days, which resulted in turbid colloidal suspension. The product was filtered off without further purification, and then immediately studied by MALDI TOF MS.

Supporting Figures and Tables

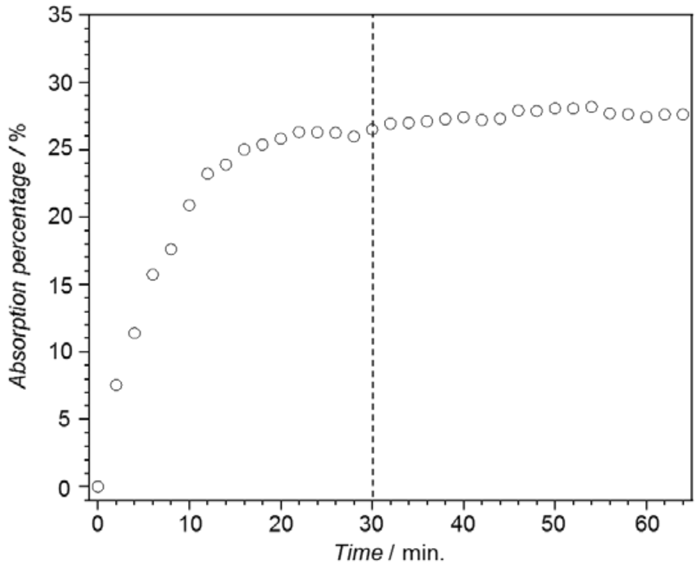

Figure S1. UV-Vis spectroscopy study of absorption percentage of monomer **1** on surfactant monolayer with respect to time. The absorption intensity of the Soret band (at 437 nm) of porphyrin units in monomer **1** was measured to investigate the absorption of monomer **1** under the SOS surfactant monolayer. Absorption percentage can be expressed by

$$Absorption\ percentage = \frac{A_0 - A}{A_0} \times 100$$

where A_0 and A represent initial absorption intensity at 437 nm and absorption intensity at 437nm, respectively. Thus, ~27% of monomer **1** was readily adsorbed underneath the SOS monolayer.

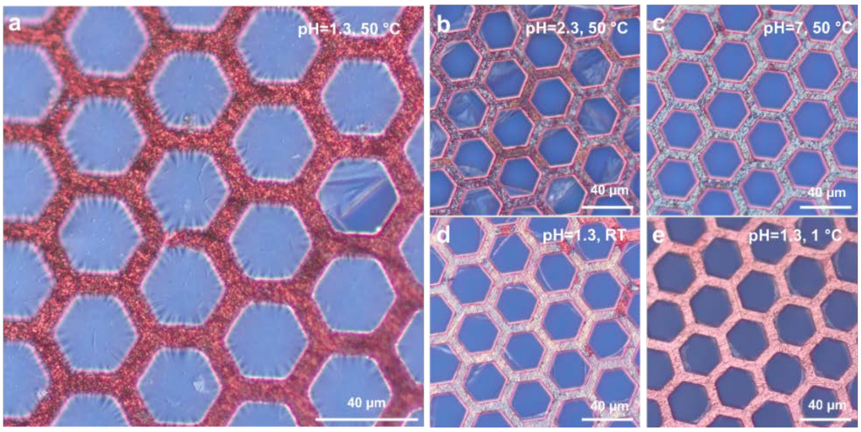

Figure S2 Temperature and pH and influence on the synthesis of **2D BECOF-PP**. a) Optical microscopy images of **2D BECOF-PP** film suspended over a copper grid, synthesized under optimized condition (pH=1.3, 50 °C, 7 days). Control conditions: b) pH=2.3, 50 °C, 7days; c) pH=7, 50 °C, 7 days; d) pH=1.3, RT, 7 days; and e) pH=1.3, 1 °C, 7days. When pH was in the range of 2.3 and 7.0, we could not achieve free-standing, robust 2D BECOF-PP film possibly due to the low polymerization degree. This is in contrast to free-standing film at pH=1.3. In addition, the contrast reactions at different temperatures suggest that synthesis of 2D BECOF-PP requires elevated temperature while low temperatures such as room temperature and 1 °C are not favorable for on-water polymerization.

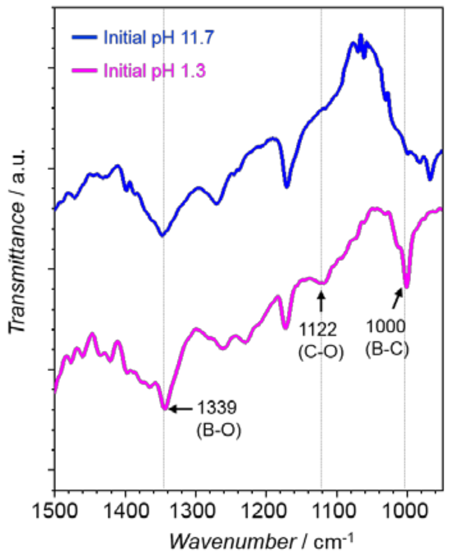

Figure S3. FT-IR of the products after reaction of monomer **1** and monomer **2** on surfactant-assisted water surface under acidic or basic condition. Vibrations in the corresponding bands of B-O, C-O and B-C reveal the formation of C_2O_2B ring under acidic condition (pH=1.3), which confirms the formation of **2D BECOF-PP**. Whereas, the C-O band is not observable for the synthetic product under basic condition, indicating that the formation of boronate ester failed at pH=11.7.

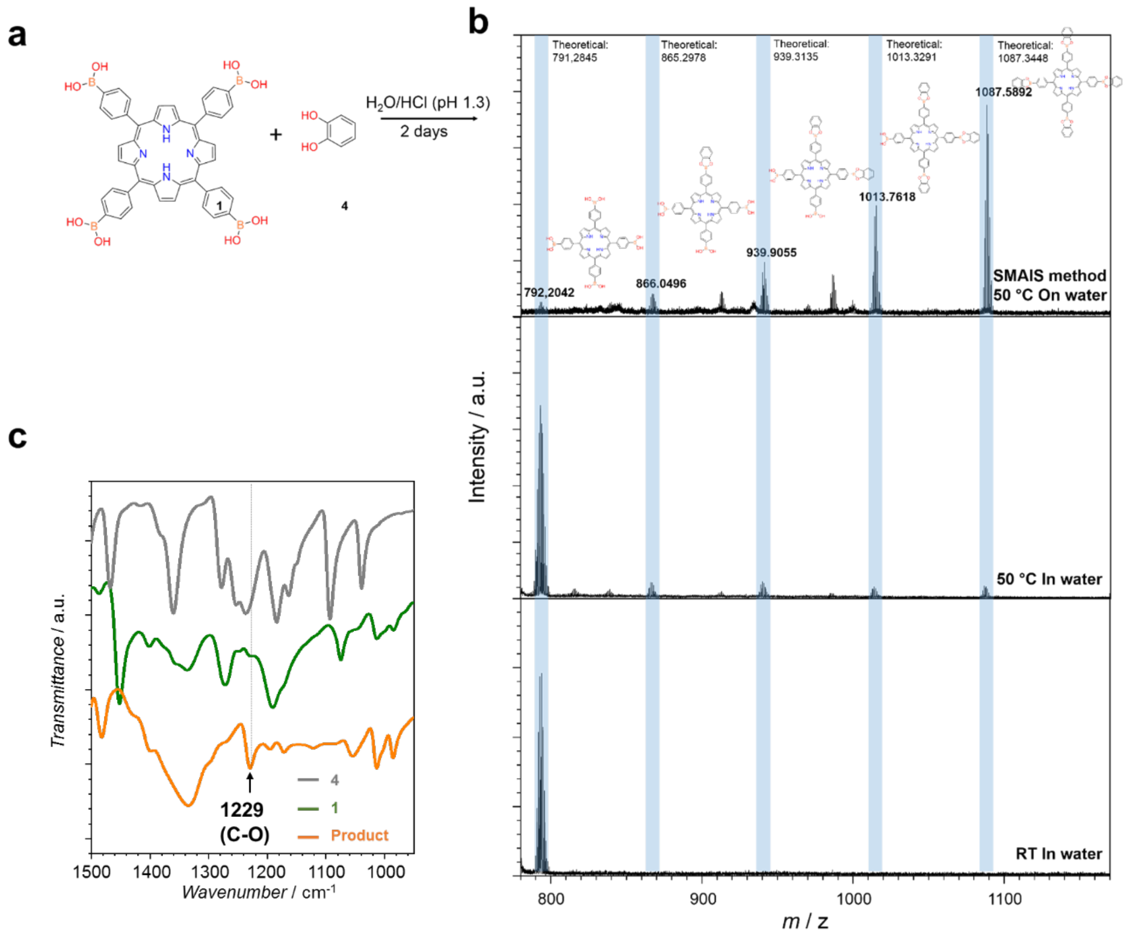

Figure S4. Model reaction between monomer **1** and 1,2-dihydroxybenzene (**4**) proves the feasibility of the formation of boronate ester linkage on water. a) Reaction scheme and employed monomers. b) MALDI-TOF MS measurement of solid products, which shows a matching isotopic pattern starting at m/z = 866.0496, 939.9055, 1013.7618 and 1087.5892 in agreement respectively with mono, bis, tris and tetrakis boronate ester bonding. (Calculated $[M+1H]^+$; 865.2978, 939.3135, 1013.3291 and 1087.3448, respectively) c) FT-IR spectra of reactants (**1**, **4**) and product of model reaction. C−O stretching mode at 1229 cm^{-1} is characteristic of boronate ester formation which is assembled from reactant **4**.[10]

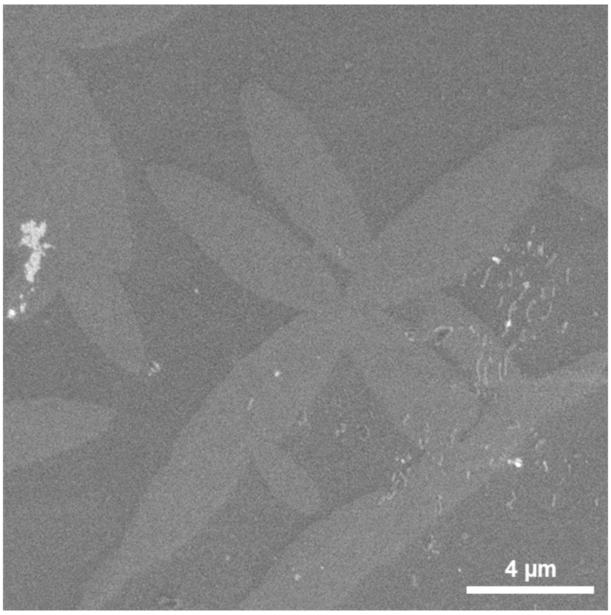

Figure S5. SEM image of **2D BECOF-PP** film.

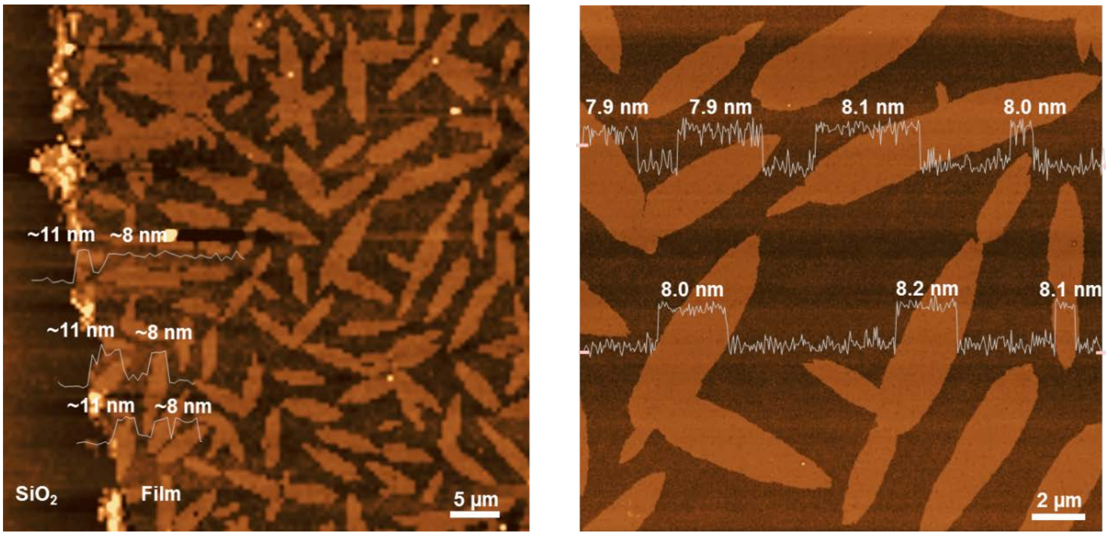

Figure S6. AFM images of **2D BECOF-PP** film. The average roughness on a domain surface is 0.84 nm.

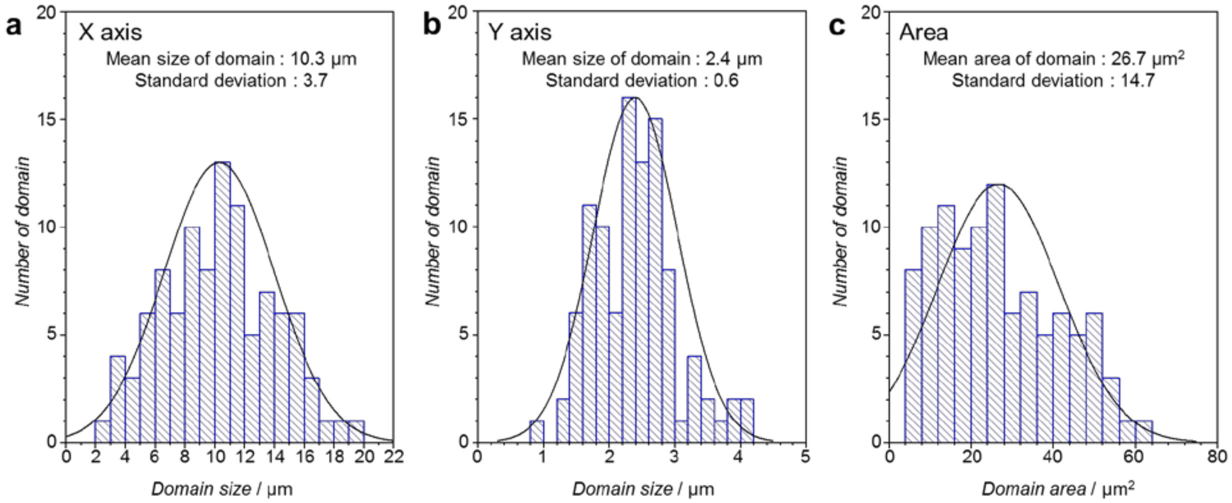

Figure S7. Domain size distribution of a) x axis (Longer horizontal direction of domain) b) y axis (shorter vertical direction of domain) and c) domain area.

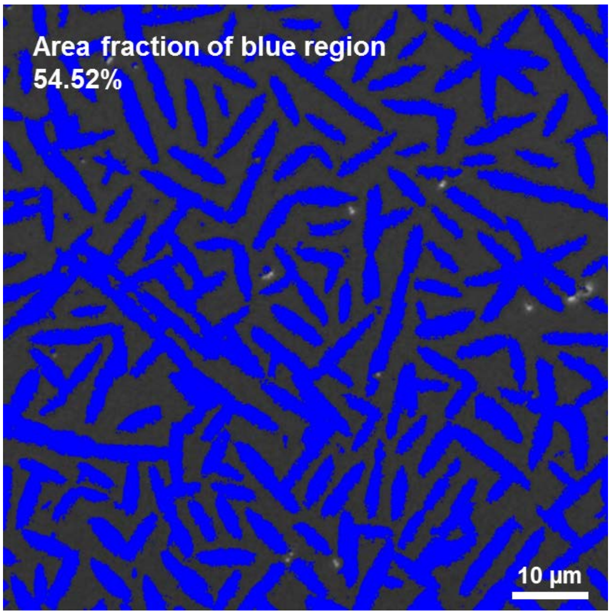

Figure S8. Area fraction of **2D BECOF-PP** crystal domains was analyzed by optical microscope image, using image processing software (ImageJ). First the image was thresholded, in order to produce a binary image (blue and dark grey), and then the area fraction of blue region was calculated. It shows an area ratio of crystalline domains in the whole film as 54.52%

a b
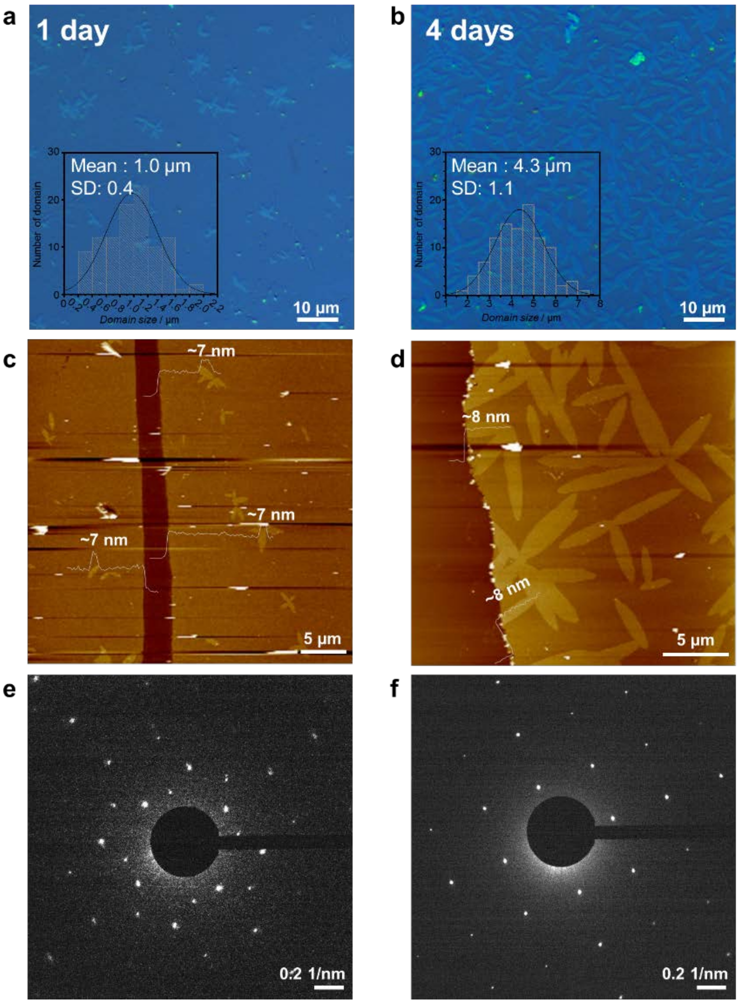

Figure S9. 2D BECOF-PP domains grown after different reaction time. Optical microscopy images of **2D BECOF-PP** films suspended over a Si/SiO_2 substrate with different reaction time: a) 1 day; b) 4 days; Thickness of the domains respect to reaction time: c) 1 day; f) 4 days; SAED revealed that domains are crystalline structure after reaction time e) 1 day and f) 4days.

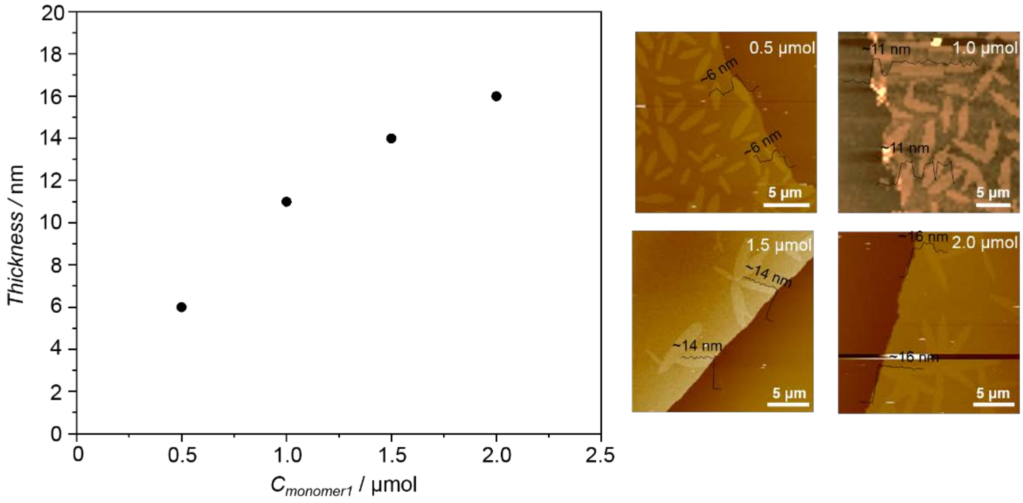

Figure S10. Thickness of **2D BECOF-PP** with respect to concentration of monomer **1**.

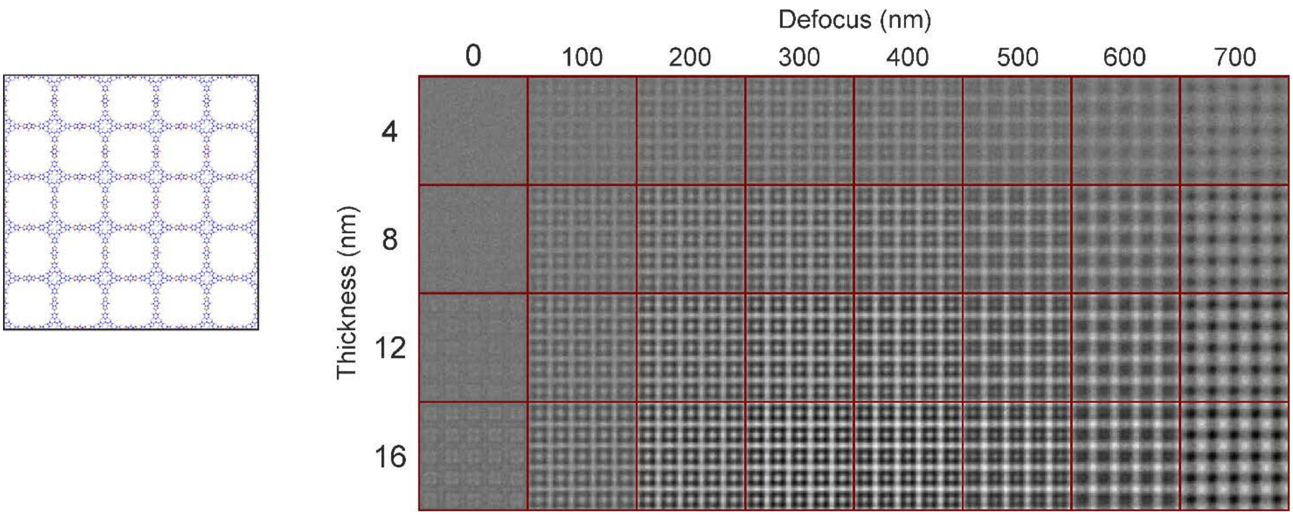

Figure S11. Simulated thickness-defocus map of AA-stacked **2D BECOF-PP** in (001) projection.

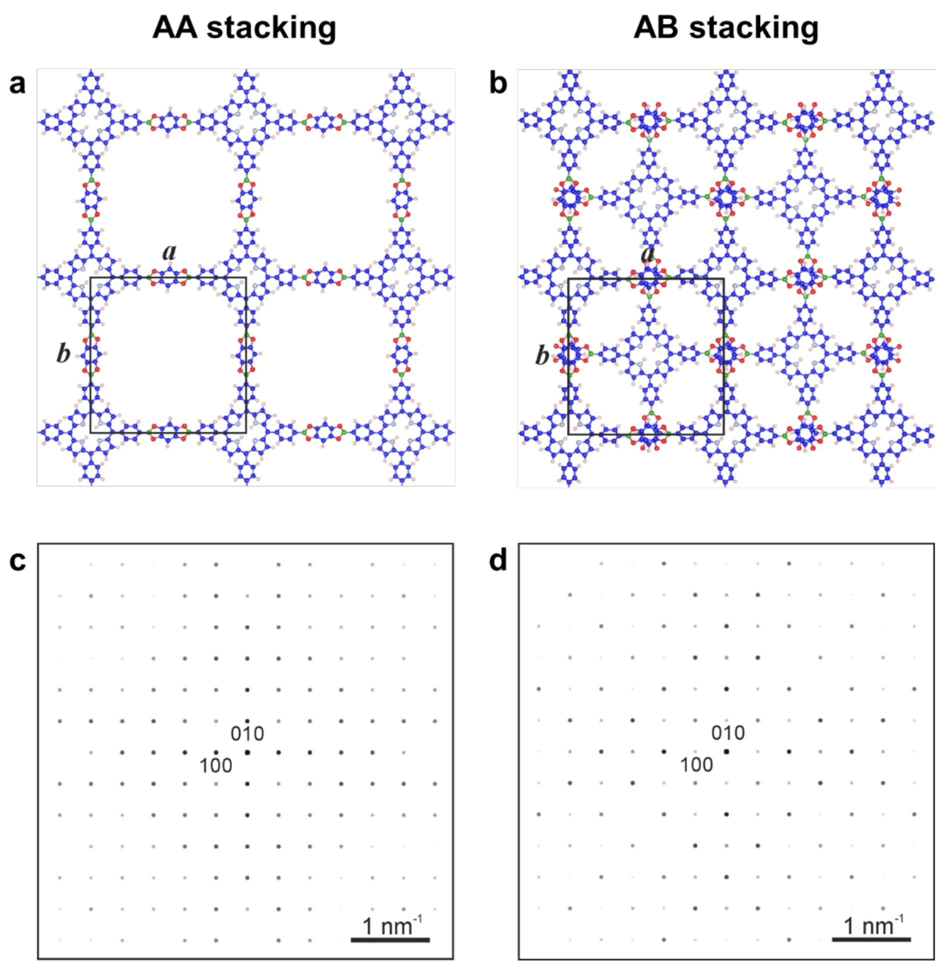

Figure S12. Structural models of **2D BECOF-PP** with an a) eclipsed structure (AA stacking), and b) staggered structures (AB stacking). c-d) Simulated electron diffraction patterns from structures (a-b), respectively.

Table S1. Comparison of d values obtained by DFT calculation and SAED for **2D BECOF-PP**

Reflection (hkl)	*d* by DFT (Å)	*d* =1/*r*[a] by SAED (Å)
(100)	24.90	24.6
(200)	49.80	49.2
(010)	24.90	24.6

[a] ***r*** ; reciprocal space d-spacing.

Table S2. Lattice parameters of different stacking modes calculated by DFT for **2D BECOF-PP**. Stacked structures are modelled as a bilayer and therefore, the interlayer distance is half of c.

	AA-stacking	AB-stacking
a	24.90	24.94
b	24.90	24.94
c	7.6	6.4
α	90	90
β	90	90
γ	90	90

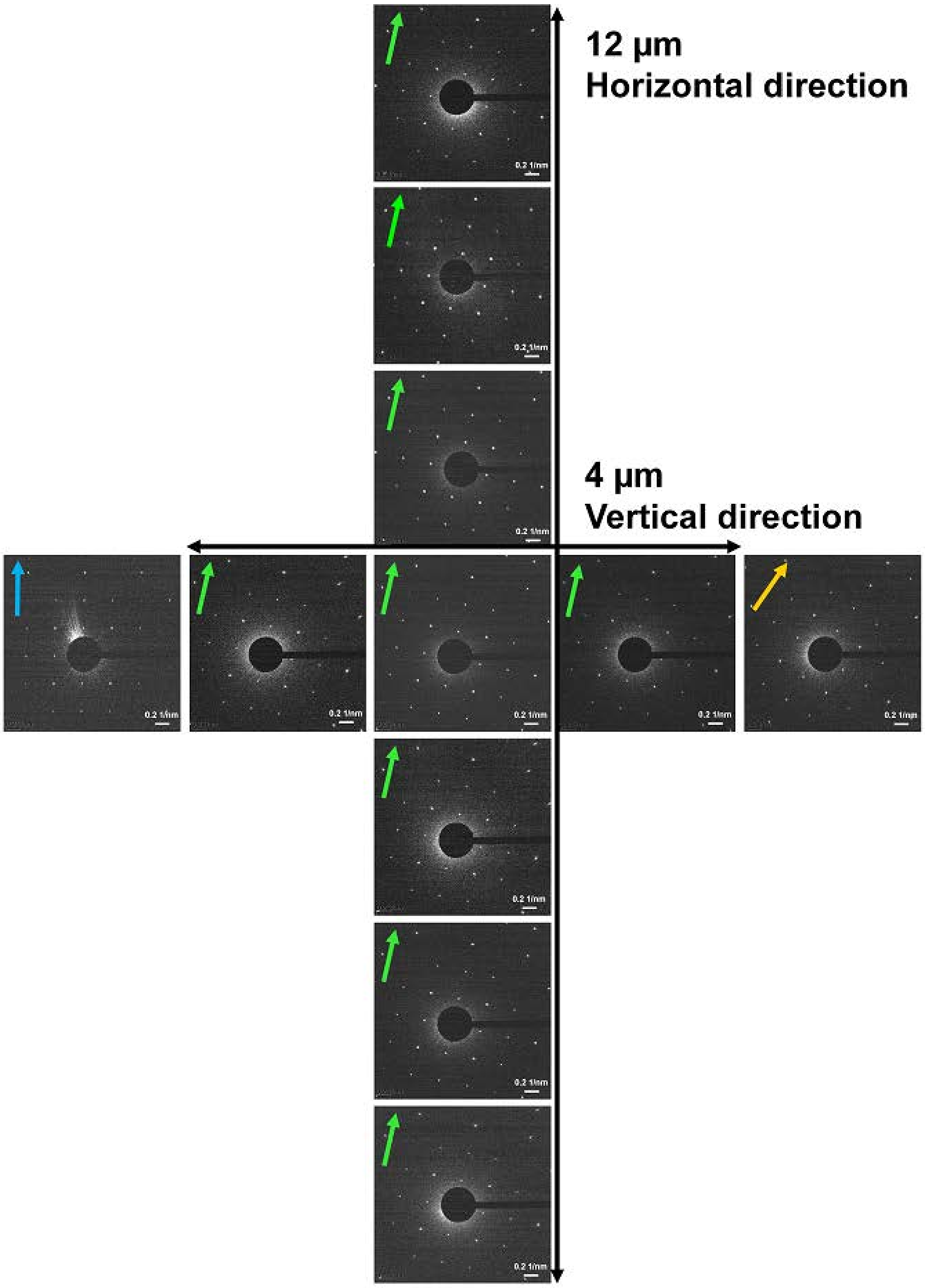

Figure S13. SAED patterns of **2D BECOF-PP** with identical lattice orientation (green arrows) across one domain revealing single-crystalline feature (size ~ 50 μm^2). Each diffraction pattern was achieved 2 μm apart.

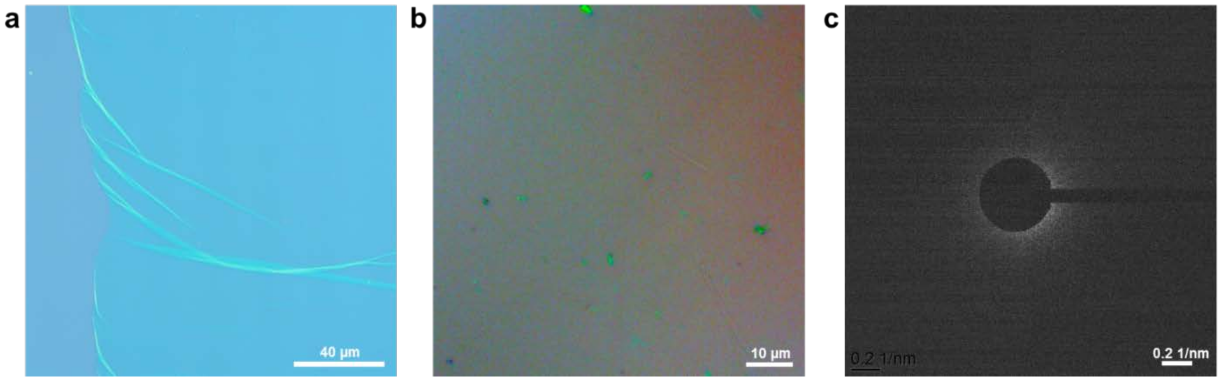

Figure S14. 2D BECOF-PP film synthesized without surfactant monolayer by the identical reaction protocol. a) Optical microscopy image of film suspended over a SiO_2 substrate shows smooth and wrinkled surface. b) Polarized microscopy image of film, no appearance of optically observable anisotropic grain. c) SAED patterns shows amorphous structure.

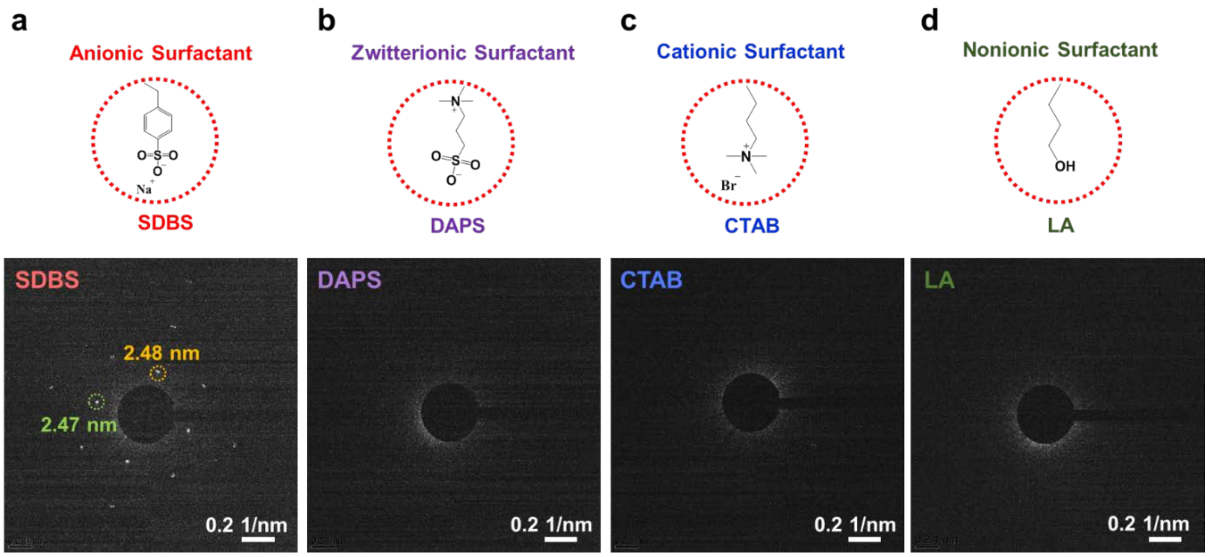

Figure S15. Contrast experiments for exploring the influence of various surfactants on the synthesis of crystalline **2D BECOF-PP** film. The employed surfactants include a) anionic surfactant (sodium dodecylbenzenesulfonate, **SDBS**), b) zwitterionic surfactant (3-(Decyldimethylammonio)propanesulfonate, **DAPS**), c) cationic surfactant (cetrimoniumbromide, **CTAB**), and d) nonionic surfactant (lignoceryl alcohol, **LA**). Among them, **SDBS** functions similar to **SOS** which can guide the self-assembly of monomer **1** via electrostatic interaction between sulfate groups and protonated porphyrin macrocycles, thus leading to the formation of **2D BECOF-PP** crystals. As contrast, the other surfactants only resulted in amorphous film, which can be attributed to the poor ordering of monomers under surfactant monolayer due to the electrostatic repulsion.

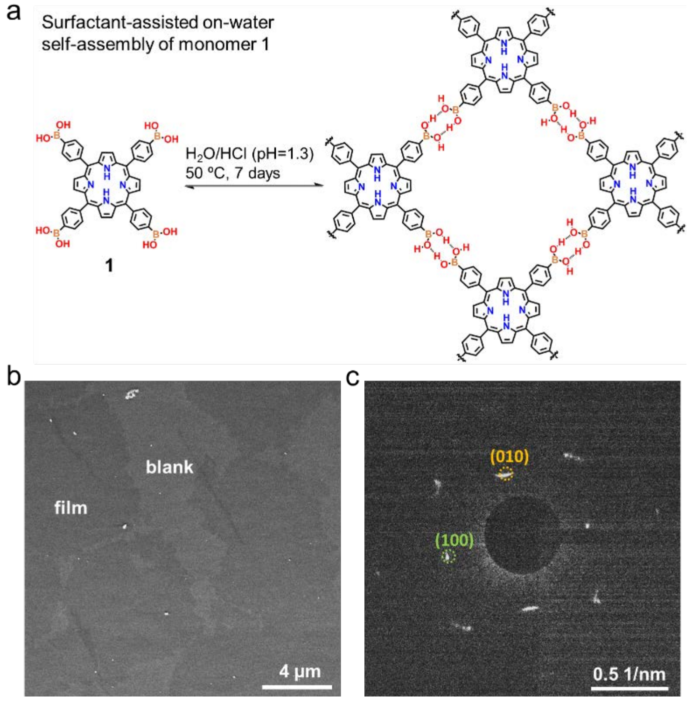

Figure S16 a) Reaction scheme and chemical structure of self-assembled film of 1 by SMAIS. b) SEM image of self-assembled film of 1 on a SiO_2 substrate. Self-assembled film is not robust and a few cracks are evident. c) Experimental electron-diffraction pattern of self-assembled film of 1, the green and yellow circles indicate the (100) and (010) planes, respectively.

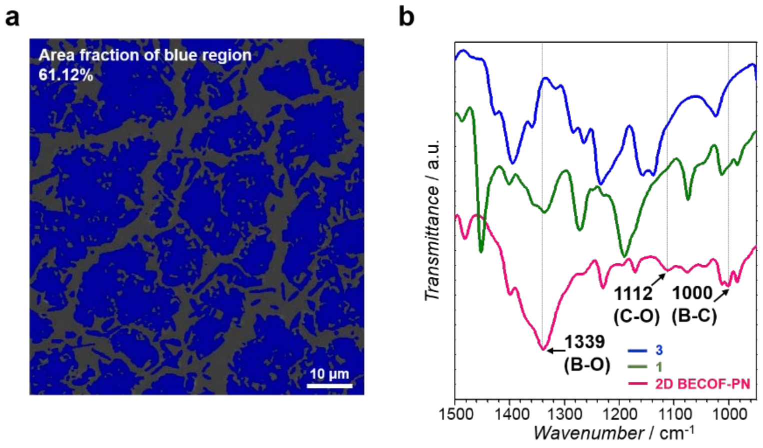

Figure S17. a) Area fraction of **2D BECOF-PN** crystal domains was analyzed by optical microscope image, using image processing software (ImageJ). First the image was thresholded, in order to produce a binary image (blue and dark grey), and then the area fraction of blue region was calculated. It shows an area ratio of crystalline domains in the whole film as 61.12%. b) FT-IR spectra of monomers (**1**, **3**) and **2D BECOF-PN**.

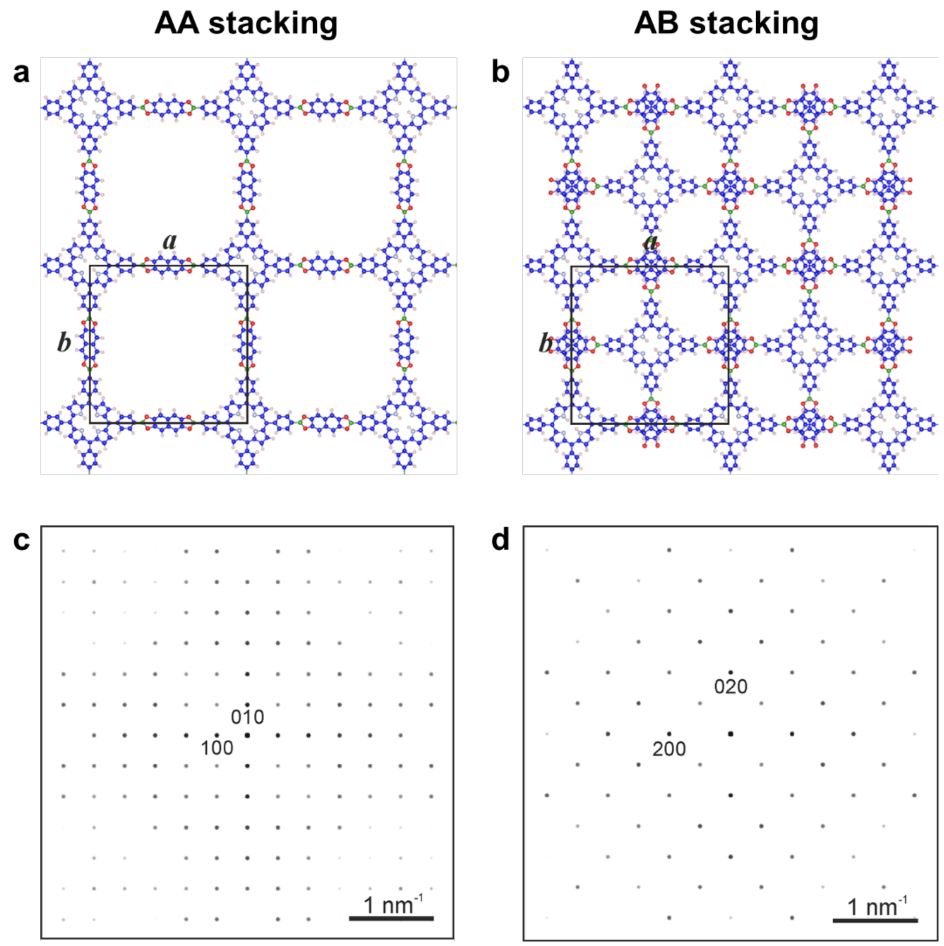

Figure S18. Structural models of **2D BECOF-PN** with an a) eclipsed structure (AA stacking), and b) staggered structures (AB stacking). c-d) Simulated electron diffraction patterns from structures (a-b), respectively.

Table S3. Comparison of d values obtained by DFT calculation and SAED for **2D BECOF-PN**

**Reflection (hkl)**	***D* by DFT (Å)**	***d* =1/*r*[a] by SAED (Å)**
(100)	27.32	26.0
(200)	54.64	52.1
(010)	27.32	26.0

[a] ***r*** ; reciprocal space d-spacing.

Table S4. Lattice parameters of different stacking modes calculated by DFT for **2D BECOF-PN**. Stacked structures are modelled as a bilayer and therefore, the interlayer distance is half of c.

	**AA-stacking**	**AB-stacking**
a	27.32	27.48
b	27.32	27.48
c	7.6	6.6
α	90	90
β	90	90
γ	90	90

Table S5. Lattice parameters of monolayer calculated by DFT for Self assembly of monomer **1**, **2D BECOF-PP** and **2D BECOF-PN**.

	Self assembly of monomer **1** **(Å)**	**2D BECOF-PP (Å)**	**2D BECOF-PN (Å)**
a	22.808	25.15	27.61
b	22.804	25.15	27.61

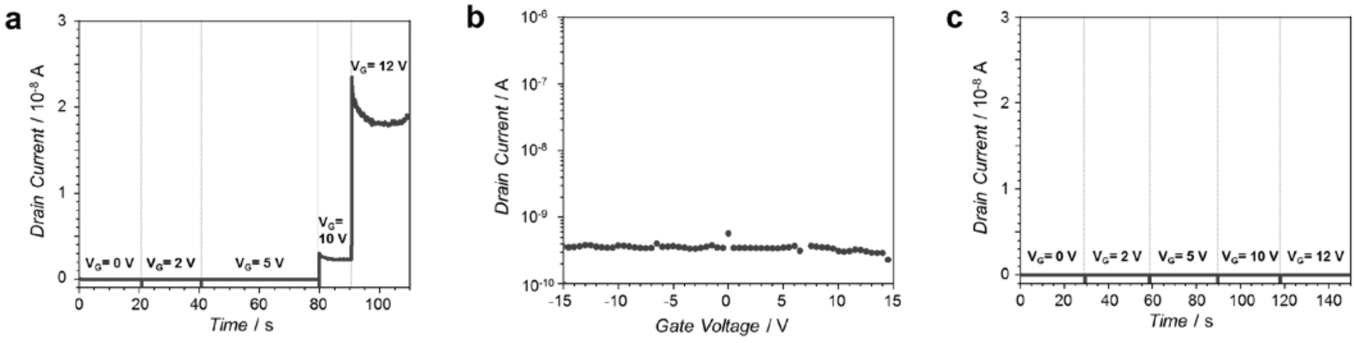

Figure S19. a) Drain current change upon increasing gate voltage of Bare SiNW device. b) Transfer characteristics and c) Drain current change upon increasing gate voltage of Bare **2D BECOF-PP** film. e) Learning pulse example of 5 V with 1000 ms period and 500 ms duty cycle.

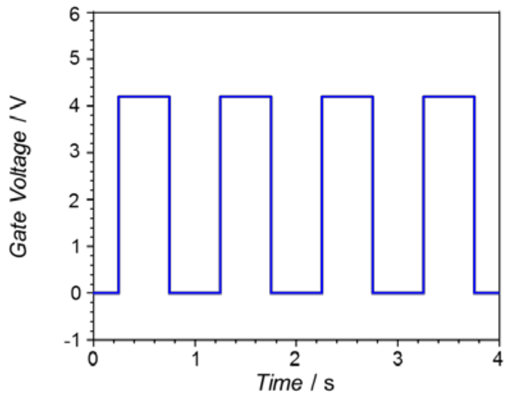

Figure S20. Learning pulse example of 5 V with 1000 ms period and 500 ms duty cycle.

References

[1] J. Hutter, M. Iannuzzi, F. Schiffmann, J. VandeVondele, *WIREs Comput. Mol. Sci.* **2014**, 4, 15-25.
[2] J. P. Perdew, A. Ruzsinszky, G. I. Csonka, O. A. Vydrov, G. E. Scuseria, L. A. Constantin, X. Zhou, K. Burke, *Phys. Rev. Lett*. **2008**, 100, 136406
[3] S. Grimme, J. Antony, S. Ehrlich, H. Krieg, *J. Chem. Phys*. **2010**, 132, 154104
[4] T. Rim, K. Kim, S. Kim, C. Baek, Member, M. Meyyappan, Y. Jeong, J. Lee, *IEEE Electron Device Letters*, **2013**, 34, 8, 1059-1061
[5] B. Ibarlucea, T. Rim, C. Baek, J. de Visser, L. Baraban, G. Cuniberti, *Lab Chip*, **2017**, 17, 4283-4293
[6] X. Feng, L. Chen, Y. Dong, D. Jiang, *Chem. Commun*. **2011**, 47, 1979-1981
[7] S. Wan, F. Gándara, A. Asano, H. Furukawa, A. Saeki, S. K. Dey, L. Liao, M. W. Ambrogio, Y. Y. Botros, X. Duan, S. Seki, J. F, Stoddart, O. M. Yaghi, *Chem. Mater*. ***2011***, 23, 4094-4097
[8] J. Moussa, V. Gandon, M. N. Rager, M. Malacria, L. Chamoreau, H. Amouri, *Eur. J. Inorg. Chem*. **2009**, 3703-3707
[9] J. Hellberg, E. Dahlstedt, M. E. Pelcman, *Tetrahedron* **2004**, 60, 40, 8899-8912
[10] M. K. Smith, B. H. Northrop, *Chem. Mater*. **2014**, 26, 3781-3795.
